# RegionGraph: Region-Aware Graph-Based Building Reconstruction from Satellite Imagery

**DOI:** 10.3390/jimaging12040161

**Published:** 2026-04-08

**Authors:** Lei Li, Chenrong Fang, Wei Li, Kan Chen, Baolong Li, Qian Sun

**Affiliations:** 1 School of Electronic and Information Engineering, Nanjing University of Information Science and Technology, Nanjing 210044, China; 2 College of Intelligence and Computing, Tianjin University, Tianjin 300072, China; 3 Infocomm Technology Cluster, Singapore Institute of Technology, Singapore 138683, Singapore

**Keywords:** structural reconstruction, remote sensing image reconstruction, primitive detection, region-aware, subgraph solving, graph contraction

## Abstract

Structural reconstruction helps infer the spatial relationships and object layouts in a scene, which is an essential computer vision task for understanding visual content. However, it remains challenging due to the high complexity of scene structural topologies in real-world environments. To address this challenge, this paper proposes RegionGraph, a novel method for structural reconstruction of buildings from a satellite image. It utilizes a layout region graph construction and graph contraction approach, introducing a primitive (layout region) estimation network named ConPNet for detecting and estimating different structural primitives. By combining structural extraction and rendering synthesis processes, RegionGraph constructs a graph structure with layout regions as nodes and adjacency relationships as edges, and transforms the graph optimization process into a node-merging-based graph contraction problem to obtain the final structural representation. The experiments demonstrated that RegionGraph achieves a 4% improvement in average F1 scores across three types of primitives and exhibits higher regional completeness and structural coherency in the reconstructed structure.

## 1. Introduction

Building structure extraction from top-down remote sensing imagery [1] has been an active research topic in both the computer vision and remote sensing communities. Buildings are key objects that convey rich geographic information in remote sensing images, and their extraction is important for many applications, such as land cover classification [2], urban planning [3], and geographic information database updating. In the context of smart city development, accurate and automated building extraction is particularly important, as it directly affects the efficiency and reliability of downstream applications. Structural information extracted from remote sensing images provides valuable support for urban planning, land resource management, and real estate registration. Moreover, such information promotes the intelligent use of remote sensing data and improves its practical value. In military scenarios, remote sensing imagery can also be used to analyze the structure and layout of buildings, providing useful information for reconnaissance tasks.

**Figure 1:**
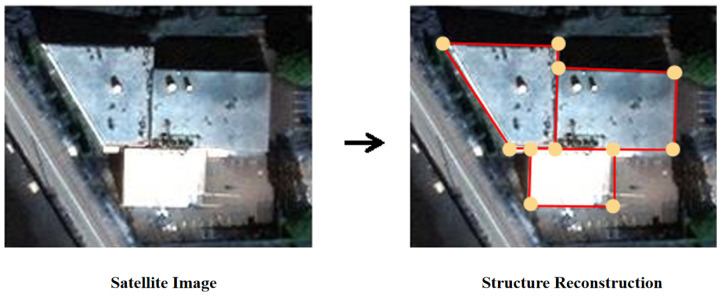
Example of extracting building structures from remote sensing images [1], the right image shows the reconstructed structure with corners and edges highlighted.

Despite its importance, building structure extraction from remote sensing images remains challenging. A major difficulty lies in handling building layouts with arbitrary and complex topologies. In particular, for outdoor building vectorization from satellite images [1], as illustrated in Figure 1, the traditional Manhattan-world assumption often fails due to long-range imaging effects and perspective distortion. These factors make accurate structure extraction more difficult. Therefore, developing an accurate, efficient, and automated method for building structure extraction from remote sensing images is of significant practical value.

Early building reconstruction methods for remote sensing imagery mainly relied on heuristic algorithms [4,5]. Although effective, these methods often had a high computational cost. With the rapid development of deep neural networks (DNNs), remote sensing image processing has undergone significant progress. DNNs [6] show strong performance in detecting low-level primitives, such as corner points, greatly improving detection accuracy and efficiency.

However, despite these advances, understanding high-level geometric structures, such as global topology and graph relationships, remains challenging for DNN-based methods. Most state-of-the-art approaches still rely on traditional optimization techniques to infer high-level structures after low-level primitives are detected. While these optimization methods are effective, they often involve complex formulations and extensive engineering to encode structural constraints, resulting in complicated and time-consuming pipelines. Therefore, improving high-level structural reasoning while maintaining efficient low-level primitive detection remains an open problem in building reconstruction from remote sensing imagery.

To address these challenges, this paper proposes a structured reconstruction framework based on graph construction and graph optimization for building structure extraction from remote sensing images. The core idea is to use a graph model to represent and integrate topological information in a scene, and to iteratively refine this representation through optimization. Based on this framework, we introduce a new method, RegionGraph. The experimental results show that the proposed method achieves competitive performance on building structure extraction tasks. Compared with traditional heuristic approaches and DNN-based low-level primitive detection methods, RegionGraph provides more accurate structural reconstruction and demonstrates higher robustness when handling complex scenes and topologies. Our main contributions are summarized as follows:

- We propose a structured reconstruction framework based on graph construction and graph optimization. In the graph construction stage, topological information is encoded into an initial graph representation. In the graph optimization stage, this representation is refined by automatically adjusting graph nodes and edges to improve reconstruction accuracy and efficiency.
- We introduce RegionGraph for building structure extraction from overhead remote sensing images. The method incorporates a primitive estimation network, ConPNet, which uses regional heatmap context to estimate primitive locations and attributes. By representing building topology as a region-based graph and formulating graph optimization as a node-merging contraction process, RegionGraph improves both regional completeness and structural consistency.
- We conduct comparison and ablation experiments on the SpaceNet dataset. The results demonstrate that the proposed method performs well in terms of regional completeness and structural relationship modeling, achieving performance comparable to state-of-the-art methods.

Note that we compare RegionGraph with a set of representative methods that span the major paradigms in building structure extraction from remote sensing imagery. Given the large body of existing work, these baselines are not intended to be exhaustive, but are selected to reflect commonly adopted reconstruction approaches. Through this comparison, we demonstrate that explicitly incorporating region-aware representations together with graph-based structural optimization leads to improved structural completeness and topological consistency. Notably, the proposed region-level graph formulation is method-agnostic and can be readily integrated into other primitive-based or wireframe reconstruction pipelines, highlighting its general applicability beyond the specific instantiation used in RegionGraph.

The remainder of this paper is organized as follows. Section 2 reviews related work on building structure extraction from remote sensing images. Section 3 describes the proposed RegionGraph method in detail. Section 4 presents the experimental results, including comparison and ablation studies, as well as qualitative visualizations. Finally, Section 5 concludes the paper and discusses future research directions.

## 2. Related Work

### 2.1. Structural Reconstruction and Structural Reasoning

Structural reconstruction belongs to the broader domain of structural reasoning, together with tasks such as human pose estimation [7] and semantic relational reasoning in images [8]. These tasks share a common goal of extracting vectorized structural representations from input data. Depending on the reconstruction target, such representations may include line segments [9], planar surfaces [10,11,12], room layouts [13,14], and polygonal rings [15].

Research on building extraction from remote sensing images can be traced back to the 1990s [16]. Early approaches mainly treated building extraction as a pixel-level image segmentation problem. However, such methods often produce jagged, irregular, and fragmented building boundaries, which are insufficient for practical applications. To address this issue, vector-based reconstruction methods were introduced, where buildings are represented as closed polygonal rings. More recently, reconstruction methods have further shifted their focus toward modeling internal and finer-grained structural details in order to obtain more accurate and complete building vector representations. The increasing complexity and variability of building topologies make building structure extraction from remote sensing images both challenging and impactful.

### 2.2. Traditional Structural Reconstruction Methods

Traditional structural reconstruction methods mainly rely on basic image processing techniques, such as histograms [17], Hough transforms, superpixel segmentation, and geometric fitting. For example, Okorn et al. [17] detect vertical planes in 3D point clouds by constructing histograms of vertical point distributions to generate floor plans. Furukawa et al. [10] and Silberman et al. [11] perform planar reconstruction using graph-cut formulations. Cabral et al. [18] recover floor layouts through dynamic programming, while Delage [19] estimates room layouts using Bayesian networks.

These traditional approaches rely heavily on heuristic rules and handcrafted assumptions, making them sensitive to noise and complex scenes. As a result, their performance often degrades when applied to real-world data with irregular structures. With the development of neural networks, structural reconstruction has gradually shifted toward data-driven solutions, enabling more robust modeling of complex structural patterns.

### 2.3. Deep Learning for Building Extraction from Remote Sensing Images

In recent years, deep learning-based methods have significantly improved the accuracy of building extraction from remote sensing images. Early approaches based on Convolutional Neural Networks (CNNs) [6] and Fully Convolutional Networks (FCNs) [20] typically formulate building extraction as a semantic segmentation problem, where each pixel is assigned a category label [21,22,23]. While these methods can identify building regions, they are unable to distinguish individual building instances.

To overcome this limitation, instance segmentation methods such as Mask R-CNN [24], SOLO [25], YOLACT [26], and PANet [27] have been applied to building extraction tasks. These methods predict individual building masks using bounding boxes or prototype-based representations. However, instance segmentation methods still face challenges, such as interference between overlapping instances and inaccurate boundary localization [28]. Moreover, both semantic and instance segmentation approaches produce pixel-level outputs that require extensive post-processing and still fall short of manual-level boundary delineation.

### 2.4. Contour-Based and Topology-Aware Methods

Another line of research treats building extraction as a contour regression problem, where the vertex coordinates of building polygons are directly predicted [29,30,31,32,33,34]. Compared to pixel-based segmentation methods, contour-based approaches are theoretically more efficient because they directly generate vector representations and reduce the need for post-processing steps such as raster-to-vector conversion.

However, simple contour representations often fail to capture complex building shapes, especially in scenarios where building blocks are fragmented, overlapping, or vertically varied. To address these limitations, recent studies have begun to explore the internal topology of building structures. For example, Zhang et al. [35] proposed a convolutional message-passing neural network that models buildings as structured graphs and extracts vector representations of building planar components from satellite images. This work highlights the importance of topological reasoning and graph-based modeling in structured reconstruction.

### 2.5. Summary

Despite recent progress, reconstructing building exterior structures remains challenging due to the presence of arbitrary and complex graph topologies. First, neural network-based heuristic optimization methods often require substantial computational resources, as they rely on iterative optimization over large search spaces. For complex building layouts, this process can become computationally expensive and time-consuming.

Second, end-to-end reconstruction pipelines may struggle to produce compact and well-closed structural representations. Although these methods can directly generate reconstruction results from input images, the diversity and complexity of building structures may lead to redundant or overly complex outputs. Therefore, achieving a balance between reconstruction accuracy, structural completeness, and computational efficiency remains a key research direction in structured building reconstruction from remote sensing imagery.

Recent transformer-based contour and graph reconstruction models further explore global attention mechanisms. RegionGraph differs by emphasizing region-aware graph initialization and contraction for structural compactness.

## 3. Method

### 3.1. Overall Architecture

**Figure 2:**
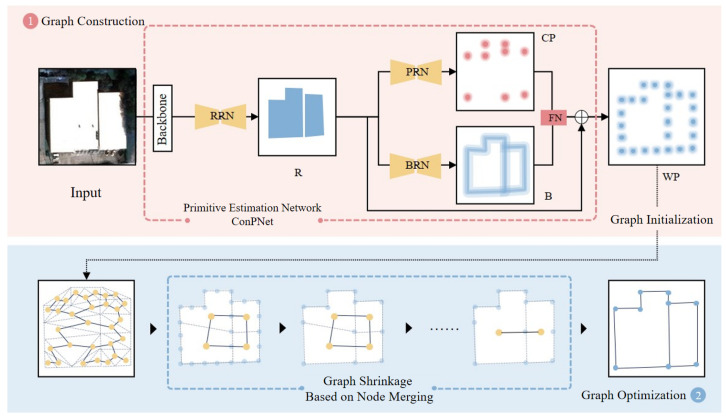
Overall architecture of RegionGraph. The method consists of two stages: (1) graph construction, where ConPNet predicts structural primitive heatmaps (region, boundary, corner, and sampled points) from the input satellite image and initializes a region-based topological graph; (2) graph optimization, where node merging is applied to contract and refine the graph structure, producing the final vectorized building reconstruction.

The overall architecture of the proposed RegionGraph is illustrated in Figure 2.

**Figure 3:**
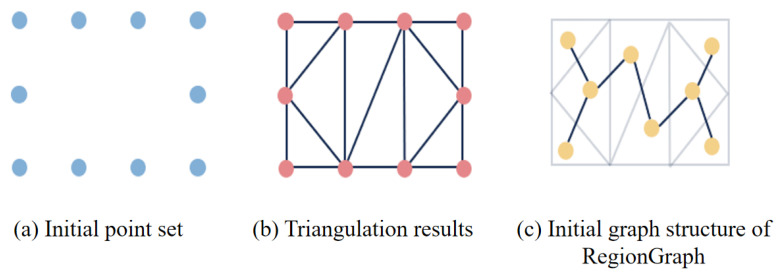
Methods for constructing graph structures. (**a**) Sampled contour points predicted by ConPNet are first used to generate a triangulated partition of the building footprint. (**b**) Each triangular unit is treated as a graph node, and adjacency between neighboring triangles is encoded as graph edges. (**c**) This process converts low-level geometric primitives into a structured topological graph, which serves as the input to the subsequent graph contraction stage.

The method represents the vectorized reconstruction result as a region-based topological graph, where nodes correspond to region (room-like) primitives and edges encode adjacency relationships between neighboring regions, as shown in Figure 3. This graph representation provides an explicit and structured description of building topology.

**Figure 4:**
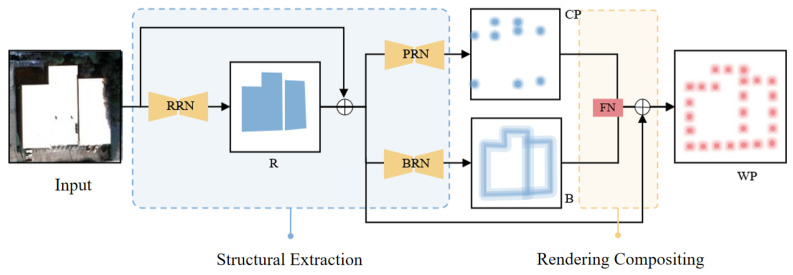
Overall architecture of the proposed ConPNet. ConPNet contains a structure extraction module composed of three reconstruction branches, the Region Reconstruction Network (RRN), Boundary Reconstruction Network (BRN), and Point Reconstruction Network (PRN), followed by a Fusion Network (FN) for sampled-point heatmap synthesis. The predicted region (R), boundary (B), and corner (CP) heatmaps provide structural cues that are integrated to produce the sampled-point heatmap (WP), which serves as the basis for graph initialization.

RegionGraph follows a two-stage pipeline. In the first stage, contour sampling points are obtained through primitive extraction. Based on these sampled points, a triangulation process is applied, and each triangulated unit is treated as a node to construct an initial graph structure. This step transforms low-level geometric primitives into a graph representation that preserves local spatial relationships. Figure 4 details the internal architecture of ConPNet used in the first stage.

In the second stage, a node-merging strategy is applied to the initial graph to perform graph contraction. By iteratively merging nodes according to predefined criteria, the graph is simplified and refined, leading to the final reconstruction result. This region-based graph construction and optimization strategy effectively improves both the regional completeness and the structural accuracy of building extraction from remote sensing images.

### 3.2. Graph Construction: ConPNet

We present the proposed ConPNet, a novel contextual information-based primitive estimation network. In the graph construction stage, RegionGraph first initializes the graph by estimating keypoint information through a keypoint heatmap regression task. For building structure extraction from remote sensing images, structural lines correspond to the contours of building roof components. Therefore, ConPNet focuses on regional features of building rooftops and extracts keypoint information based on the contextual information provided by regional heatmaps. The predicted geometric primitives at keypoint locations form the basis for subsequent topological graph construction. These keypoints include corner points and sampling points, which correspond to contour intersection points and uniformly distributed samples along contour edges, respectively.

ConPNet performs the transformation from remote sensing images to building structural primitive heatmaps, which can be viewed as an image-to-image translation task under a constrained scenario. Given a remote sensing image *X* containing a building, the image includes intrinsic structural information that remains invariant under different environmental conditions. This structural information is denoted as *S*:

(1) $$S=\left\{E,V\right\},\;V=\left\{(x,y)|x,y\in S\right\},\;E=\left\{\left(v_{i},v_{j}\right)|v_{i},v_{j}\in V\right\}$$

Here, *V* denotes the set of building corner points, and *E* denotes the set of contour edges. The combination of *V* and *E* uniquely determines the building structure. Since corners and edges belong to different geometric categories, we adopt a sampling-based representation to unify these primitives and approximate *S* using $S^{\prime }$:

(2) $$S^{\prime }=\left\{P\right\},\;P=\left\{(x,y)|(x,y)\;\mathrm{on}\;e,\;e\subseteq E\right\}$$

Here, *P* represents the set of sampling points uniformly distributed along building contour edges, and V⊆P. Therefore, for a given input image *X*, the task of ConPNet can be expressed as:

(3) $$R\left(S^{\prime }\right)=F\left(X\right)$$

Here, F(X) denotes the heatmap extraction operation, and $R(S^{\prime })$ represents the kernelized heatmap generated from the point set $S^{\prime }$. Based on this formulation and the structural properties of different heatmaps, ConPNet decomposes the extraction of $S^{\prime }$ into two stages: structural extraction and rendering synthesis, defined as:

(4) $$\begin{array}{cc} F(X) & =F_{f}\left(F_{ve}\left(X\right)\right) \end{array}$$

(5) $$\begin{array}{cc} F_{ve}\left(X\right) & =[F_{v}\left(X\right),F_{e}\left(X\right)] \end{array}$$

1. **Structure extraction operation $F_{ve}$:** This step aims to remove environmental and appearance variations while recovering vectorized structural information. Specifically, corner reconstruction $F_{v}$ and edge reconstruction $F_{e}$ are applied to suppress background noise and façade textures, and to recover building contour corners and edges. The outputs of $F_{v}$ and $F_{e}$ are concatenated along the channel dimension before being passed to the subsequent rendering synthesis module.
2. **Rendering synthesis operation $F_{f}$:** This step performs discrete sampling of boundary structures based on detected corner points. Sampling points are generated along boundary heatmaps according to corner locations to produce the final sampled-point representation.

Since building reconstruction from remote sensing images emphasizes the main building region, and because direct extraction of corners and contours is challenging, ConPNet incorporates regional geometric priors into the structure extraction process. The transformation is therefore expressed as:

(6) $$F\left(X\right)=F_{f}\left(F_{ve}\left(F_{r}\left(X\right)\right)\right),$$

where $F_{r}$ denotes the regional heatmap reconstruction operation.

Based on this design, ConPNet consists of two main components: a structural heatmap reconstruction network and a rendering synthesis network. The structural heatmap reconstruction network includes a **Region Reconstruction Network (RRN)**, a **Boundary Reconstruction Network (BRN)**, and a **Point Reconstruction Network (PRN)**, which correspond to $F_{r}$, $F_{e}$, and $F_{v}$, respectively. **The Fusion Network (FN)** implements the rendering synthesis operation $F_{f}$. The separation of region-, boundary-, and corner-aware branches is motivated by their distinct structural roles in graph initialization and contraction.

Note that all reconstruction modules in ConPNet, including the RRN, BRN, PRN, and FN, are trained end-to-end using supervised heatmap regression losses. No predefined operators or handcrafted rules are used in the structure extraction or rendering synthesis stages.

#### 3.2.1. Structural Design of ConPNet

As illustrated in Figure 4, ConPNet adopts a two-module architecture composed of the structure extraction module and the rendering synthesis module. The structure extraction module processes the input image to generate multiple primitive heatmaps, while the rendering synthesis module integrates these primitives to produce the final sampled point heatmap.

**Figure 5:**
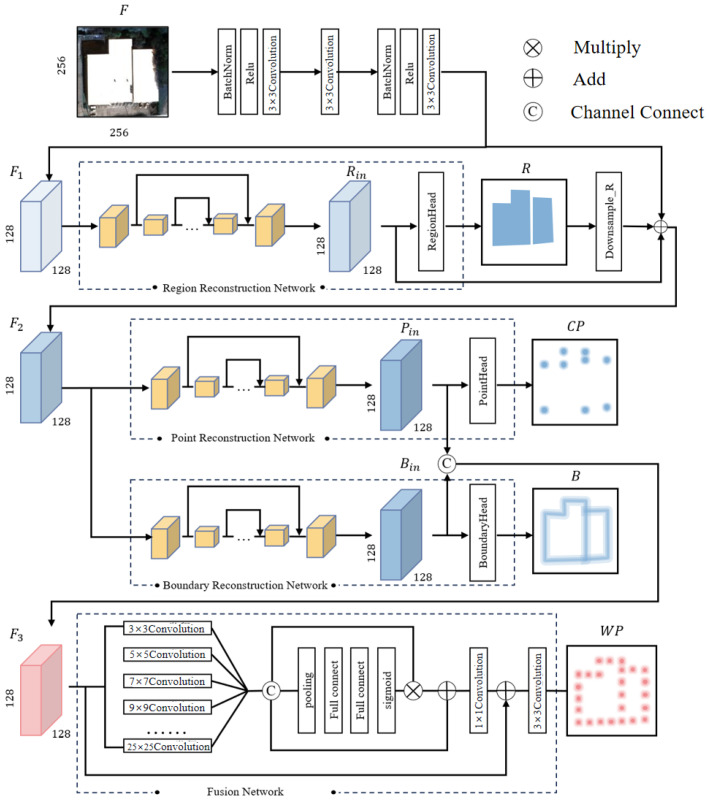
Detailed architecture of the proposed ConPNet.

The detailed architecture of each module is shown in Figure 5. Given a remote sensing image $F\in R^{H\times W\times 3}$, a backbone network first extracts low-level features, producing a feature map $F_{1}\in R^{\frac{H}{2}\times \frac{W}{2}\times N}$. The backbone consists of a 3×3 convolution layer, a residual ConvBlock, and another 3×3 convolution layer. The extracted feature map $F_{1}$ is then fed into the RRN to obtain intermediate region-aware features $R_{in}$ and the corresponding regional heatmap *R*.

The regional heatmap *R* is upsampled and combined with $F_{1}$ and $R_{in}$ through residual fusion to produce $F_{2}$, which serves as the input to both the PRN and the BRN. These two networks generate corner-aware features $P_{in}$ and edge-aware features $B_{in}$, respectively, which are further transformed into the corner heatmap *P* and the boundary heatmap *B*. Finally, $P_{in}$ and $B_{in}$ are concatenated along the channel dimension to form $F_{3}$, which is passed to the Fusion Network to produce the sampled-point heatmap WP. While channel attention improves feature weighting during fusion, alternative lightweight fusion strategies may also be explored in future work.

The following subsections describe the structure extraction and rendering synthesis modules in detail.

#### 3.2.2. Structure Extraction

The goal of the structure extraction stage is to generate multiple primitive heatmaps that encode building structural information. Since Hourglass networks [36] have demonstrated strong performance in multi-scale feature extraction and localization tasks [37], we adopt Hourglass-style architectures as the backbone of the structure extraction module. The parameter settings of the base convolutional blocks are summarized in Table 1, and the configurations of selected network modules are shown in Table 2.

The RRN aims to suppress complex background information and extract region-aware features corresponding to the building body. It consists of a three-layer Hourglass network [36] followed by a RegionHead. The Hourglass network produces a region-aware feature map $R_{in}$, which is further transformed by the RegionHead to generate the regional heatmap *R*. This heatmap serves as a geometric prior that constrains subsequent primitive heatmap generation. Due to the smooth spatial distribution of region heatmaps, the RRN is trained using a mean-squared-error loss. The configuration of RegionHead is presented in Table 2.

The PRN infers the spatial distribution of corner points along building contours. Similarly to the RRN, the PRN adopts a three-layer Hourglass architecture followed by a PointHead. The fused feature map incorporating regional priors is processed to produce corner-aware features and the corresponding corner heatmap *P*. The PRN is trained using an $L_{1}$ loss to encourage accurate localization of corner points. The parameter settings of the PointHead are provided in Table 2.

The BRN is designed to recover building contour boundaries from the fused feature representation. It shares the same Hourglass-based architecture as the PRN and outputs an edge heatmap *B* through a BoundaryHead. To handle the imbalance between boundary and non-boundary pixels, the BRN is trained using the Adaptive Wing Loss [37]. The detailed configuration of BoundaryHead is also shown in Table 2.

#### 3.2.3. Rendering Compositing

The rendering compositing stage aims to generate the sampled-point heatmap by discretely sampling boundary information guided by corner cues. This process is implemented by the Fusion Network (FN), which takes corner-aware and edge-aware feature maps as inputs, and then outputs the sampled-point heatmap WP.

**Figure 6:**
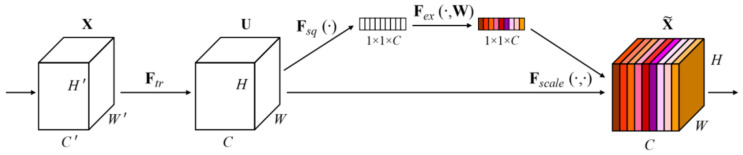
Squeeze–Excitation module based on channel attention.

To effectively fuse multi-scale structural information, the FN incorporates a channel attention mechanism. Channel attention assigns adaptive weights to feature channels, enabling the network to emphasize informative channels while suppressing less relevant ones. As illustrated in Figure 6, the channel attention module follows a Squeeze–Excitation–Transform pipeline, where global average pooling is used for feature squeezing, fully connected layers model inter-channel dependencies, and a sigmoid activation normalizes the channel weights.

Specifically, the FN applies multiple convolutional layers with different kernel sizes (from 3×3 to 25×25) to capture both local and global structural information. The resulting multi-scale features are concatenated and passed through the channel attention module to produce scale-adaptive feature weights. The weighted features are then fused through element-wise multiplication and addition, followed by a convolution layer to generate the final sampled-point heatmap WP.

The Fusion Network is trained using a combination of Adaptive Wing Loss [37] and $L_{1}$ loss:

(7) $$L_{wp}=\lambda_{1}L_{AWL}+\lambda_{2}L_{1}.$$

Here, $y_{wp}$ denotes the ground-truth sampled-point heatmap and ${\hat{y}}_{wp}$ denotes the predicted heatmap. The Adaptive Weight Loss balances different supervision terms using learnable or predefined scaling factors. The weights $\lambda_{1}$ and $\lambda_{2}$ balance the contributions of the corresponding loss terms. They were selected based on validation-set experiments to ensure stable training and balanced performance across region, boundary, and corner reconstruction tasks and performance. In all experiments, the parameters ω, ϵ, α, θ, $\lambda_{1}$, and $\lambda_{2}$ were set to 14, 1, 2.1, 0.5, 0.5 and 0.5, respectively.

### 3.3. Graph Optimization: Graph Shrinkage via Node Merging

This section introduces the second stage of RegionGraph, namely graph optimization. The initial region-based graph constructed from ConPNet outputs is simplified into a target graph that represents complete building components.

The optimization stage in RegionGraph is formulated as a geometric simplification problem over a triangulated planar graph rather than as a relational representation learning task. Since the initial graph is constructed by triangulating sampled contour points, over-segmentation naturally appears at the primitive level. Node-merging-based graph contraction is therefore structurally aligned with this initialization: it consolidates adjacent triangular regions into coherent roof components while preserving planarity and connectivity. Compared with learning-based graph optimization methods such as graph neural networks, or global partitioning techniques such as spectral clustering and min-cut formulations, our approach emphasizes deterministic, interpretable, and computationally efficient structural refinement without introducing additional learnable parameters at the optimization stage.

**Figure 7:**
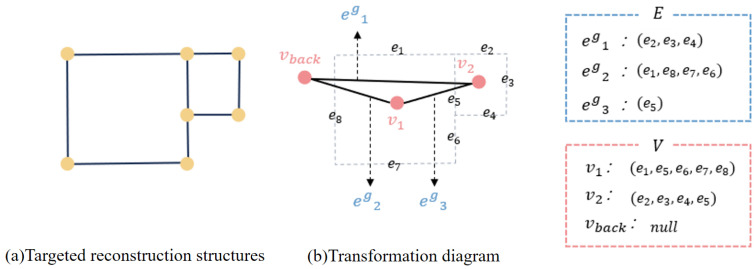
Graph structure representation of reconstruction results. (**a**) Example of a target building reconstruction composed of multiple connected structural components. (**b**) Corresponding transformation into a region-based graph representation. Each structural region is modeled as a graph node, and shared boundaries between regions are encoded as relational edges. The sets *E* and *V* illustrate how edges and nodes are organized in the graph structure, including the background node $v_{back}$. This representation forms the basis for the subsequent graph contraction process.

Given an initial graph G={E,V}, where *V* denotes nodes and *E* denotes edges, the optimization objective is to minimize the following energy function:

(8) $$E_{graph}=E_{node}\left(V\right)+E_{edge}\left(E\right),$$

where the two terms measure node completeness and inter-region relationships, respectively, as shown in Figure 7.

The energy formulation focuses on enforcing local region completeness and adjacency consistency. Higher-order global topological constraints, such as cycle consistency or global connectivity regularization, are not explicitly encoded in the current model. In practice, the planar triangulation initialization and adjacency-preserving contraction implicitly maintain structural validity for footprint-level reconstruction. However, incorporating explicit global constraints may further improve robustness in highly complex building layouts.

**Node term ($E_{node}$).** The node term evaluates whether a node corresponds to a complete building roof component. It combines the regional heatmap *R* and boundary heatmap *B* to measure the structural completeness of each polygonal node:

(9) $$E_{node}\left(V\right)=\sum_{v\in V}\left(\sum_{e_{j}\in v}E_{heat}\left(e_{j}\right)\right)/v_{num}.$$

The edge heat penalty is defined as:

(10) $$E_{heat}\left(e_{j}\right)=\frac{1}{|e_{j}|}\int_{0}^{1}\left(R\left(e_{j}\left(u\right)\right)-B\left(e_{j}\left(u\right)\right)\right)\;du,$$

(11) $$e_{j}\left(u\right)=u\;e_{j}^{1}+\left(1-u\right)\;e_{j}^{2},$$

where $|e_{j}|$ is the edge length and $e_{j}^{1},e_{j}^{2}$ are its endpoints. Smaller values of $E_{heat}$ indicate a higher likelihood that an edge corresponds to a true structural boundary. Minimizing $E_{node}$ encourages each node to represent a complete building component. In implementation, the continuous integral in Equation (10) is approximated by uniform discrete sampling along each edge. Specifically, points are sampled at one-pixel intervals along the edge segment, and bilinear interpolation is used to obtain heatmap values at subpixel locations. The integral is then computed as the average of sampled values multiplied by the edge length.

**Edge term ($E_{edge}$).** The edge term measures region merging based on adjacency relationships. A small average $E_{heat}$ along a shared boundary indicates a true structural separation, while a large value suggests that two regions belong to the same component. The formulation is:

(12) $$E_{edge}=\sum_{e^{g}\in E}\left(\sum_{e_{j}\in e^{g}}E_{heat}\left(e_{j}\right)\right)/e_{num}^{g},$$

where $e^{g}$ denotes a relational edge and $e_{num}^{g}$ is the number of triangular edges associated with it. Minimizing $E_{edge}$ reduces unnecessary region merging and preserves structural independence.

Overall, minimizing $E_{graph}$ is formulated as a graph contraction problem based on node merging, where regions corresponding to the same building component are progressively merged.

#### Node Merging Strategy

The goal of graph contraction is to reduce the number of nodes and edges while preserving graph connectivity. The contraction process consists of two stages:

**Triangular-region merging.** Each triangular node must belong to the same component as at least one neighboring node. To reduce the search space, nodes connected by the longest edge in each triangle are merged. Given an initial graph G={E,V}, the merged graph $G^{\prime }=\left\{E^{\prime },V^{\prime }\right\}$ is defined as:

(13) $$G^{\prime }=\left\{E^{\prime },V^{\prime }\right\},$$

(14) $$V^{\prime }=V\cup \left\{w\right\}-\left\{u,v|e^{uv}\in \left\{e_{t}^{max}\right\},\;t\in T\right\},$$

(15) $$E^{\prime }=\left\{\left(w,x\right)|\left(u,x\right)\in E,\;x\neq v\right\}\cup \left\{\left(w,y\right)|\left(v,y\right)\in E,\;y\neq u\right\},$$

where $e_{t}^{max}$ is the longest edge of triangle *t*, *T* is the triangle set, and *w* is the merged node.

**Relational-edge-based merging.** After triangular merging, polygonal nodes are further merged using relational-edge confidence. The procedure consists of three steps:

1. Compute $E_{heat}\left(e^{g}\right)$ for each relational edge $e^{g}\in E^{\prime }$.
2. Remove edges with confidence greater than a threshold $T_{merge}$:
  (16) $$E^{\prime \prime }=E^{\prime }-\left\{e^{g}|E_{heat}\left(e^{g}\right)>T_{merge}\right\}.$$
3. Merge nodes connected by the removed edges:
  (17) $$V^{\prime \prime }=V^{\prime }\cup \left\{w^{\prime }\right\}-\left\{u^{\prime },v^{\prime }|e^{u^{\prime }v^{\prime }}\in E^{\prime }-E^{\prime \prime }\right\}.$$

**Figure 8:**
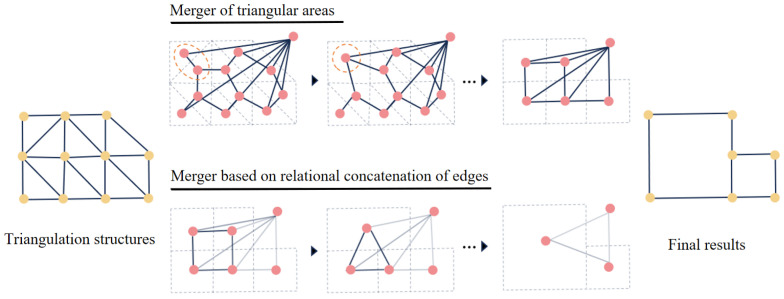
Graph shrinkage process based on node merging.

Figure 8 illustrates the overall contraction process. The initial graph is first simplified by triangular-region merging, followed by relational-edge-based merging. Finally, the dummy background node introduced during graph construction is removed to obtain the final vectorized structural representation.

## 4. Experiments and Analysis

### 4.1. Dataset and Sample Processing

#### 4.1.1. Dataset

The experiments are conducted on high-resolution satellite RGB imagery from the SpaceNet corpus [1], hosted on Amazon Web Services (AWS) as part of the SpaceNet Challenge. We adopt the benchmark dataset introduced by Nauata et al. [1], which contains 2001 building crops from three cities (Atlanta, Paris, and Las Vegas). Each image is cropped into a 256×256 RGB patch.

The dataset contains 2001 samples in total, split into 1601/50/350 for training, validation, and testing. Each sample includes a satellite RGB image and a corresponding vector-structure annotation. The vector graph represents a roof structure, where vertices correspond to corners, and edges correspond to roof components (Figure 1). Across the dataset, the average/maximum numbers of corners and edges are 12.6/93 and 14.2/101, respectively.

#### 4.1.2. Sample Processing

RegionGraph constructs graph structures based on ConPNet heatmap regression outputs. We therefore generate ground-truth heatmaps for each geometric primitive as follows:

- **Sample-point heatmap.** We uniformly sample points along each annotated edge at a 10-pixel interval to form the sampled-point set. Each sampled point is represented in the heatmap by placing a 2D Gaussian kernel centered at its pixel location, with standard deviation σ=2, resulting in the sampled-point heatmap.
- **Corner heatmap.** Corner heatmaps are generated using the same Gaussian rendering procedure applied to the annotated corner set.
- **Boundary heatmap.** Annotated edges are dilated to 3-pixel-wide line segments and then smoothed with a Gaussian filter with σ=2 to form the boundary heatmap.
- **Region heatmap.** The closed region enclosed by annotated edges is filled with 1, and all other pixels are set to 0, producing the region heatmap.

### 4.2. Evaluation Metrics and Experimental Setup

#### 4.2.1. Evaluation Metrics

We follow the evaluation protocol of Nauata et al. [1]. Metrics are computed for three primitive types (corner, edge, and region) using precision, recall, and F1.

**Corner metrics.** A predicted corner is counted as a true positive if it lies within 8 pixels of a ground-truth corner. Each ground-truth corner can match at most one prediction. The recall and precision are:

(18) $$Recall_{V}=\frac{TP_{V}}{TP_{V}+FN_{V}},\;Precision_{V}=\frac{TP_{V}}{TP_{V}+FP_{V}}.$$

**Edge metrics.** An edge is counted as correctly reconstructed if and only if both of its endpoints are correctly reconstructed. The recall and precision are:

(19) $$Recall_{E}=\frac{TP_{E}}{TP_{E}+FN_{E}},\;Precision_{E}=\frac{TP_{E}}{TP_{E}+FP_{E}}.$$

**Region metrics.** A predicted region is considered correct if its IoU with a ground-truth roof region is at least 70%. The recall and precision are:

(20) $$Recall_{R}=\frac{TP_{R}}{TP_{R}+FN_{R}},\;Precision_{R}=\frac{TP_{R}}{TP_{R}+FP_{R}}.$$

**F1 score.** We report F1 to jointly evaluate precision and recall:

(21) $$F1=\frac{2\times Recall\times Precision}{Recall+Precision}.$$

We adopt the standard SpaceNet evaluation protocol to ensure fair comparison with prior work. While corner, edge, and region F1 scores provide a widely accepted measure of reconstruction accuracy, they do not fully capture higher-order topological correctness. More explicit topology-aware metrics, such as graph edit distance or component-level IoU, could provide additional insight and are left for future investigation.

#### 4.2.2. Experimental Setup

All experiments are conducted on a server equipped with a 2.6 GHz CPU and an NVIDIA RTX 3090 GPU, the training required approximately 10 h for convergence. We implement the models in Python 3.8 with PyTorch 1.9.0. Training uses the Adam optimizer with an initial learning rate of $1\times 10^{-4}$ and weight decay of $1\times 10^{-5}$. Models are trained for 700 epochs, and the learning rate is decayed by a factor of 0.1 during the last 100 epochs. Unless specified otherwise, we set the loss weights in Equation (7) to $\lambda_{1}=0.5$ and $\lambda_{2}=0.5$, and set the graph-shrinkage threshold in Equation (16) to $T_{merge}=0.5$. The value of 0.5 was selected based on validation experiments over the range [0.3, 0.7], where region F1 peaked near 0.5, with lower values causing over-merging and higher values leading to under-merging. ConPNet consists of three Hourglass-style reconstruction branches (RRN, PRN, BRN) with shared feature width N=256, followed by a lightweight fusion module. The overall network contains trainable parameters in the order of $10^{7}$ (about 30 M), which is comparable to other multi-branch architectures for structural localization.

Although experiments are conducted on 256 × 256 patches following the SpaceNet protocol, RegionGraph can be extended to large satellite imagery using a sliding-window or tiling strategy with overlap. ConPNet is fully convolutional, allowing arbitrary image sizes during inference, and graph contraction is applied locally on initialized graph components. Therefore, the method can be integrated into large-scene processing pipelines through standard tile-based aggregation.

### 4.3. Comparative Evaluation

To validate the effectiveness of RegionGraph, we compare it against seven representative baselines: PolyRNN++ [38], PPGNet [39], Hamaguchi [40], SDSC-UNet [41], L-CNN [42], Nauata [1], and ConvMPN [35].

**Raster-based segmentation methods.** Hamaguchi [40] and SDSC-UNet [41] produce raster masks and require raster-to-vector conversion for structural evaluation.

**Vector reconstruction methods.** The remaining five methods directly predict vector structures. PolyRNN++ [38] performs contour regression, PPGNet [39] and L-CNN [42] focus on wireframe detection, Nauata [1] is the benchmark for satellite building vectorization, and ConvMPN [35] also reconstructs building structure via a graph representation.

Table 2 reports precision and recall for corners, edges, and regions. RegionGraph achieves the best region performance among all methods. This is primarily because our region-based graph construction preserves more area-level structure by triangulating sampled points into region nodes, which improves region recall. In addition, graph shrinkage via node merging explicitly enforces component completeness, improving region precision.

Methods that represent structures only as corner/edge primitives often ignore global region relationships, leading to overlaps or inconsistencies between adjacent components and weaker region metrics. Segmentation-based methods provide binary masks but are less sensitive to precise boundaries, which also limits region performance. RegionGraph is slightly behind L-CNN [42] and Nauata [1] on corner/edge metrics, which may be attributed to the heatmap regression and peak-based vector extraction, where discretization can introduce small localization errors compared to direct coordinate regression or detection.

Table 3 reports the F1 scores. RegionGraph achieves the best region F1 and the best average F1. Compared with Nauata [1], RegionGraph improves region F1 by nearly 8 points and improves the average F1 by about 4 points, indicating stronger overall structural consistency.

**Figure 9:**
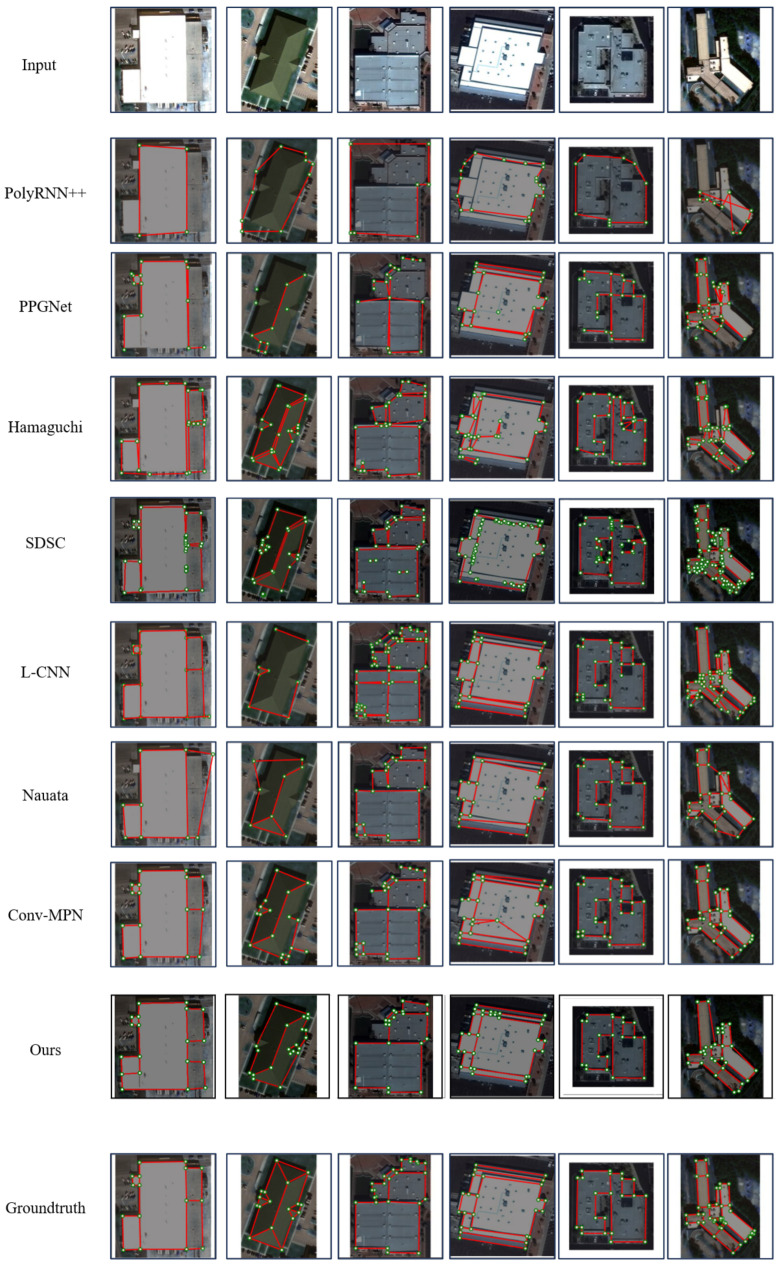
Visualization results of different methods on the SpaceNet corpus dataset.

Figure 9 presents the qualitative comparisons. RegionGraph produces more complete region structures, with fewer dangling vertices and fewer intersecting edges. PolyRNN++ [38] typically captures only the outermost contour and can miss internal structure. Primitive-based methods often suffer from fragmentation, producing isolated corners and edges. Segmentation-derived vector contours can be ambiguous at shared boundaries, where adjacent regions are represented by duplicated edges, reducing compactness. The Nauata baseline [1] relies on handcrafted constraints and is more sensitive to Manhattan layouts; in non-Manhattan cases it can show noticeable angular bias. Overall, RegionGraph achieves strong region completeness with clearer structural relationships.

**Figure 10:**
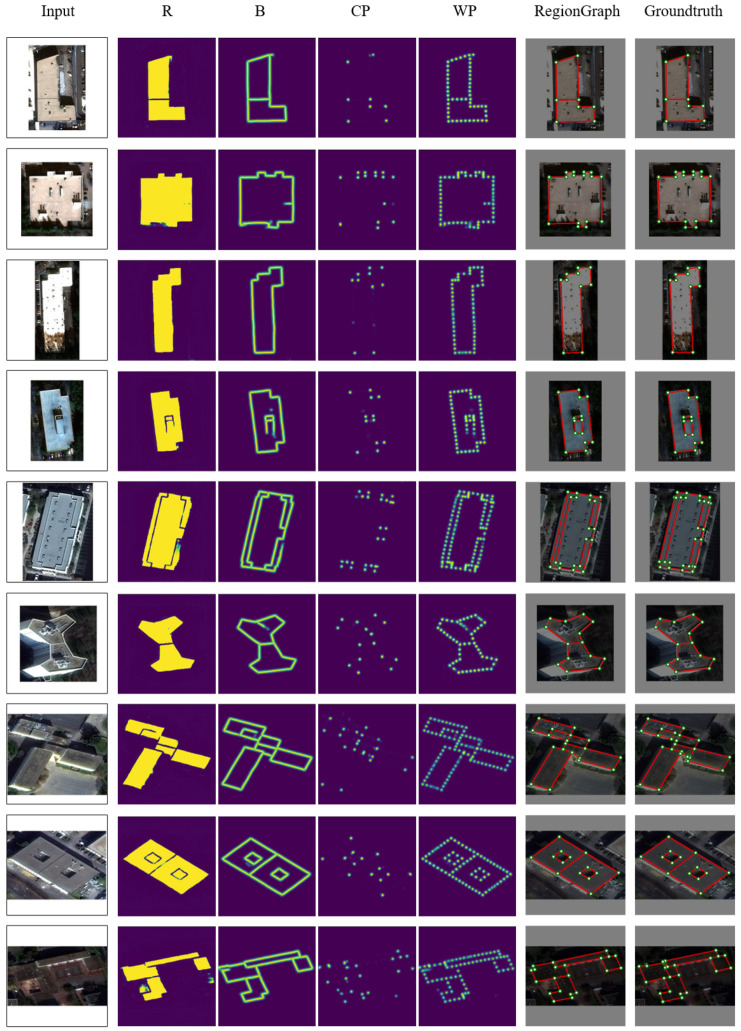
Primitive heatmaps and reconstructed structures produced by RegionGraph on SpaceNet.

Figure 10 visualizes intermediate heatmaps and final reconstructions. Representative examples of the predicted region, boundary, corner, and sampled-point heatmaps are shown in Figure 10, illustrating the behavior of the learned operators on sample images. In the graph construction stage, ConPNet predicts region (*R*), boundary (*B*), corner (CP), and sampled-point (WP) heatmaps. The region heatmap captures footprint completeness but is less sharp at boundaries, while boundary and corner heatmaps refine geometric details. The sampled-point heatmap integrates these cues to support graph initialization. In the optimization stage, graph shrinkage converts the noisy initial structure into a compact vector representation and can correct local errors using global consistency. For instance, shifted corners can be corrected, false-positive internal edges can be removed, and missing internal boundaries can be recovered.

**Figure 11:**
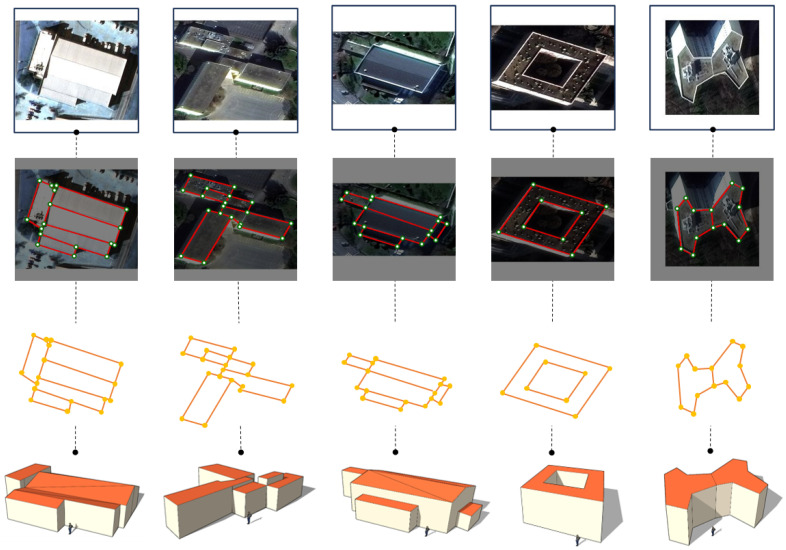
Partial 3D reconstruction results based on RegionGraph outputs. They are manually constructed from the 2D vector outputs of RegionGraph to illustrate potential downstream 3D reconstruction applications. The proposed method itself performs 2D structural reconstruction and does not directly generate 3D geometry.

To better illustrate the reconstructed structures, we manually create 3D models in SketchUp using selected RegionGraph results (Figure 11). The reconstructed floor plans remain accurate for complex composite buildings and ring structures with courtyards, providing a strong basis for downstream 3D reconstruction. Note that building height and roof type are inferred manually from shading.

While RegionGraph improves regional completeness and structural compactness, certain failure cases remain. These include over-merging in closely adjacent roof components, under-segmentation in highly fragmented structures, and occasional missing internal boundaries under weak boundary responses. These limitations reflect challenges in heatmap quality and graph initialization.

### 4.4. Ablation Study

We evaluate different module combinations to validate the RegionGraph design. Table 4 summarizes the ablation settings, and Table 2 and Table 3 report the quantitative results. Note that the ablation study is designed to evaluate representative configurations of RegionGraph rather than exhaustively testing all possible combinations. Because the graph optimization stage operates on the graph produced by ConPNet, the two stages are structurally coupled and cannot be fully disentangled without modifying the pipeline. Therefore, we adopt a progressive configuration strategy to assess the impact of the main components.

- **Point_*sample*_.** Use sampled points as the triangulation input; otherwise use corners.
- **Contract_*tri*_.** Enable triangular-region merging; otherwise remove this contraction step.
- **Contract_*heat*_.** Enable relational-edge-based merging; otherwise remove this contraction step.
- **R.** Use region heatmap prior in ConPNet; otherwise remove RegionHead and Downsample_*R*_.
- **CA.** Use channel attention in the Fusion Network; otherwise replace it with simple channel summation.

Table 2 and Table 3 show that sampled-point initialization improves structural recall compared to corner-only initialization (Setting 1 vs. Setting 3), indicating that sampled points capture richer boundary and region cues. Graph optimization substantially improves all metrics (Setting 2 vs. Setting 5), demonstrating that node-merging-based shrinkage is critical for removing redundancy and enforcing component-level consistency. The comparison among Settings 3, 4, and 5 highlights that both contraction stages matter: triangular merging provides coarse simplification, while relational-edge-based merging further refines adjacency using global cues. Incorporating region priors improves heatmap quality (Setting 5 vs. Setting 6), and channel attention further strengthens multi-scale fusion (Setting 6 vs. Setting 7).

**Figure 12:**
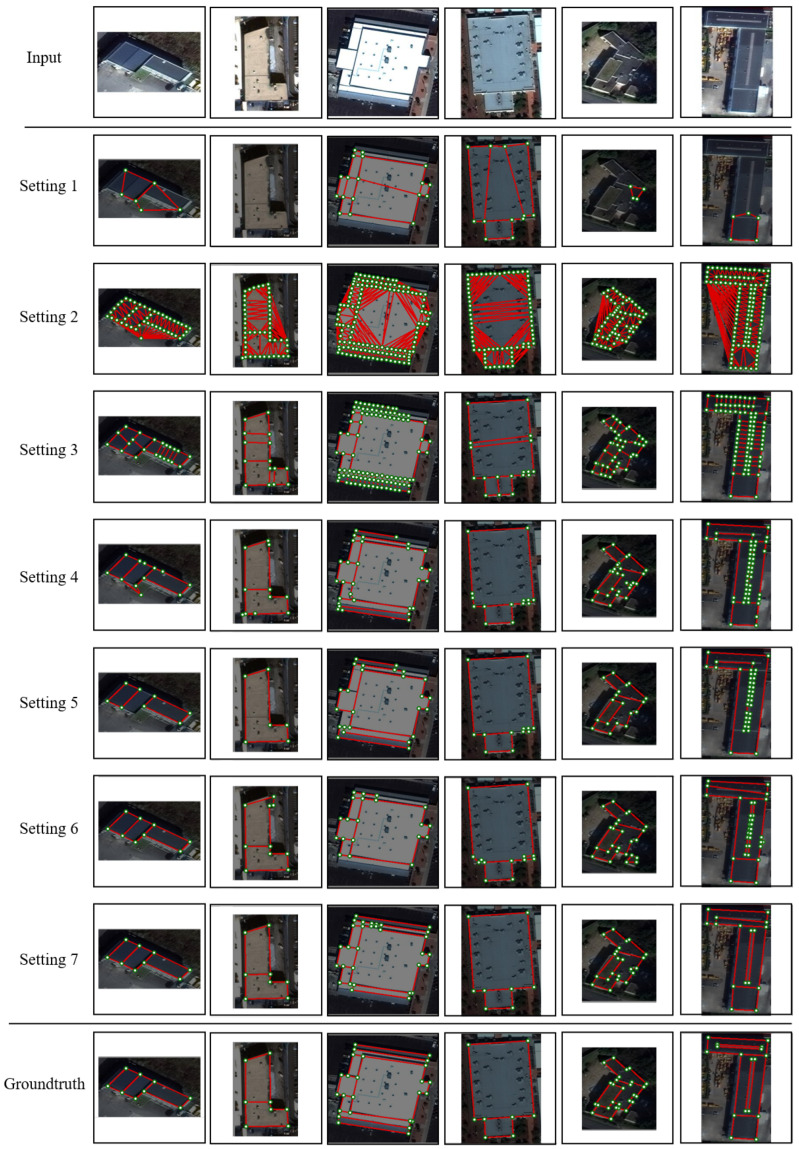
Visualization of reconstruction results under different ablation settings.

Figure 12 provides qualitative evidence consistent with the quantitative trends. Sample-point-based initialization yields more complete boundaries and regions. Triangular merging removes many redundant edges, and relational-edge-based merging further suppresses false positives and improves compactness. Adding region priors and channel attention consistently moves predictions closer to the manual annotations.

All experiments follow the official SpaceNet split with identical training protocols to ensure fair comparison. The observed improvements are consistent across multiple structural metrics.

## 5. Conclusions

We proposed RegionGraph for building structure extraction from top-down satellite imagery. RegionGraph consists of two stages: (i) ConPNet predicts sampled structural primitives via heatmap regression and initializes a region graph using triangulation; (ii) graph optimization formulates structure refinement as a graph shrinkage problem and produces compact vector representations via triangular merging and relational-edge-based merging.

We evaluate RegionGraph using standard metrics for corners, edges, and regions (precision, recall, and F1). Compared with classical wireframe extraction methods (PPGNet [39], L-CNN [42]) and representative remote-sensing baselines (PolyRNN++ [38], Hamaguchi [40], SDSC-UNet [41], Nauata [1], and ConvMPN [35]), RegionGraph achieves stronger region accuracy and better overall structural consistency. The qualitative results further show improved region completeness and clearer structural relationships. The ablation studies confirm the effectiveness of each component in the proposed pipeline.

While the proposed graph contraction strategy effectively improves region-level structural consistency, the current energy formulation primarily captures local completeness and adjacency relationships and does not explicitly model higher-order global topological constraints. Future work may explore integrating global connectivity regularization or learning-based relational refinement to enhance generalization to more complex building layouts and large-scale urban scenes.

In addition, the current evaluation relies on benchmark metrics and does not explicitly quantify graph-level topological consistency, which may be further explored using dedicated topology-aware measures. Although the threshold demonstrates stable behavior within the evaluated range on SpaceNet, adaptive threshold selection or cross-dataset validation may further improve robustness under varying building typologies and imaging conditions.

A more comprehensive combinational analysis, statistical significance testing and broader comparison with recent transformer-based models and unified runtime benchmarking, as well as analysis of large-scene runtime, memory footprint, and real-time deployment, could further strengthen the empirical validation and will be considered in future work. In another future study, we plan to integrate shadow-based height estimation, which could enable a more automated 3D reconstruction pipeline.

## Figures and Tables

**Table 1:** Parameterization of the base convolution module in ConPNet.

	$\mathrm{ConvLayer}(C_{\mathrm{in}},C_{\mathrm{out}})$	$\mathrm{DownSample}(C_{\mathrm{in}},C_{\mathrm{out}})$	$\mathrm{UpSample}(C_{\mathrm{in}},C_{\mathrm{out}})$	$\mathrm{ConvBlock}(C_{\mathrm{in}},C_{\mathrm{out}})$
Input	$H\times W\times C_{in}$	$H\times W\times C_{in}$	$H\times W\times C_{in}$	$H\times W\times C_{in}$
Structure	Conv 3×3, stride = 1	Conv 3×3, stride = 1	ConvT 3×3, stride = 2	$ConvLayer(C_{in},\frac{C_{out}}{2})$
	BatchNorm	BatchNorm	BatchNorm	$ConvLayer(C_{in},\frac{C_{out}}{4})$
	ReLU	ReLU	ReLU	$ConvLayer(C_{in},\frac{C_{out}}{4})$
				Concatenate
Output	$H\times W\times C_{out}$	$\frac{H}{2}\times \frac{W}{2}\times C_{out}$	$2H\times 2W\times C_{out}$	$H\times W\times C_{out}$

**Table 2:** Comparison of corner/edge/region precision and recall on SpaceNet. The best results are shown in **bold**, and the second-best are underlined.

Method	Corner (%)		Edge (%)		Region (%)	
	Precision	Recall	Precision	Recall	Precision	Recall
**PolyRNN++ [38]**	49.6	43.7	19.5	15.2	39.8	13.7
**PPGNet [39]**	78.0	69.2	55.1	50.6	32.4	30.8
**Hamaguchi [40]**	58.3	57.8	25.4	22.3	51.0	36.7
**SDSC-UNet [41]**	42.5	70.6	25.6	35.6	42.1	42.7
**L-CNN [42]**	66.7	**86.2**	51.0	**71.2**	25.9	41.5
**Nauata [1]**	**91.1**	64.6	**68.1**	48.0	70.9	53.1
**ConvMPN [35]**	77.9	80.2	56.9	60.7	51.1	57.6
**RegionGraph (Ours)**	80.3	75.9	61.6	58.3	**71.9**	**65.4**

**Table 3:** Comparison of F1 scores on SpaceNet. The best results are shown in **bold**, and the second-best are underlined.

Method	F1-Corner (%)	F1-Edge (%)	F1-Region (%)	F1-Average (%)
**PolyRNN++ [38]**	46.4	17.1	20.4	28.0
**PPGNet [39]**	73.3	52.8	31.6	52.6
**Hamaguchi [40]**	58.0	23.8	42.7	41.5
**SDSC-UNet [41]**	53.1	29.8	42.4	41.8
**L-CNN [42]**	75.2	59.4	31.9	55.5
**Nauata [1]**	75.6	56.3	60.8	64.2
**ConvMPN [35]**	**79.0**	58.7	54.2	64.0
**RegionGraph (Ours)**	78.0	**59.9**	**68.5**	**68.8**

**Table 4:** Ablation settings for RegionGraph (✓ indicates inclusion, / means not).

Setting Name	${\mathrm{Point}}_{\mathrm{sample}}$	${\mathrm{Contract}}_{\mathrm{tri}}$	${\mathrm{Contract}}_{\mathrm{heat}}$	R	CA
**Setting 1**	/	✓	/	/	/
**Setting 2**	✓	/	/	/	/
**Setting 3**	✓	✓	/	/	/
**Setting 4**	✓	/	✓	/	/
**Setting 5**	✓	✓	✓	/	/
**Setting 6**	✓	✓	✓	✓	/
**Setting 7**	✓	✓	✓	✓	✓

## Data Availability

Restrictions apply to the availability of these data. Data were obtained from SpaceNet and are available at https://registry.opendata.aws/spacenet/(accessed on 30 March 2026) with the permission under License CC BY-NC-SA 4.0.

## References

[1] Nauata N., Furukawa Y. (2020). Vectorizing world buildings: Planar graph reconstruction by primitive detection and relationship inference. Proceedings of the European Conference on Computer Vision.

[2] Zhao S., Tu K., Ye S., Tang H., Hu Y., Xie C. (2023). Land Use and Land Cover Classification Meets Deep Learning: A Review. Sensors.

[3] Sharifi A., Khavarian-Garmsir A.R., Allam Z., Asadzadeh A. (2023). Progress and prospects in planning: A bibliometric review of literature in Urban Studies and Regional and Urban Planning, 1956–2022. Prog. Plan..

[4] Hough P.V. (1962). Method and Means for Recognizing Complex Patterns.

[5] Zhao W., Persello C., Stein A. (2020). Building instance segmentation and boundary regularization from high-resolution remote sensing images. Proceedings of the IEEE International Geoscience and Remote Sensing Symposium.

[6] LeCun Y., Boser B., Denker J.S., Henderson D., Howard R.E., Hubbard W., Jackel L.D. (1989). Backpropagation applied to handwritten zip code recognition. Neural Comput..

[7] Toshev A., Szegedy C. (2014). Deeppose: Human pose estimation via deep neural networks. Proceedings of the IEEE Conference on Computer Vision and Pattern Recognition.

[8] Xu D., Zhu Y., Choy C.B., Li F. (2017). Scene graph generation by iterative message passing. Proceedings of the IEEE Conference on Computer Vision and Pattern Recognition.

[9] Xu Y., Xu W., Cheung D., Tu Z. (2021). Line segment detection using transformers without edges. Proceedings of the IEEE/CVF Conference on Computer Vision and Pattern Recognition.

[10] Furukawa Y., Curless B., Seitz S.M., Szeliski R. (2009). Manhattan-world stereo. Proceedings of the IEEE Conference on Computer Vision and Pattern Recognition.

[11] Silberman N., Hoiem D., Kohli P., Fergus R. (2012). Indoor segmentation and support inference from rgbd images. Proceedings of the European Conference on Computer Vision.

[12] Nguyen T., Reitmayr G., Schmalstieg D. (2015). Structural modeling from depth images. IEEE Trans. Vis. Comput. Graph..

[13] Zou C., Colburn A., Shan Q., Hoiem D. (2018). Layoutnet: Reconstructing the 3D room layout from a single rgb image. Proceedings of the IEEE Conference on Computer Vision and Pattern Recognition.

[14] Yang S.T., Wang F.E., Peng C.H., Wonka P., Sun M., Chu H.K. (2019). Dula-net: A dual-projection network for estimating room layouts from a single rgb panorama. Proceedings of the IEEE/CVF Conference on Computer Vision and Pattern Recognition.

[15] Gimenez L., Hippolyte J.L., Robert S., Suard F., Zreik K. (2015). Reconstruction of 3D building information models from 2D scanned plans. J. Build. Eng..

[16] Zhang Y. (1999). Optimisation of building detection in satellite images by combining multispectral classification and texture filtering. ISPRS J. Photogramm. Remote Sens..

[17] Okorn B., Xiong X., Akinci B., Huber D. Toward automated modeling of floor plans. Proceedings of the Symposium on 3D Data Processing, Visualization and Transmission.

[18] Cabral R., Furukawa Y. (2014). Piecewise planar and compact floorplan reconstruction from images. Proceedings of the 2014 IEEE Conference on Computer Vision and Pattern Recognition.

[19] Delage E., Lee H., Ng A.Y. (2006). A dynamic bayesian network model for autonomous 3D reconstruction from a single indoor image. Proceedings of the IEEE Conference on Computer Vision and Pattern Recognition.

[20] Long J., Shelhamer E., Darrell T. (2015). Fully convolutional networks for semantic segmentation. Proceedings of the IEEE Conference on Computer Vision and Pattern Recognition.

[21] Girard N., Smirnov D., Solomon J., Tarabalka Y. (2021). Polygonal Building Extraction by Frame Field Learning. Proceedings of the IEEE/CVF Conference on Computer Vision and Pattern Recognition.

[22] He J., Cheng Y., Wang W., Ren Z., Zhang C., Zhang W. (2024). A Lightweight Building Extraction Approach for Contour Recovery in Complex Urban Environments. Remote Sens..

[23] Li K., Liu R., Cao X., Bai X., Zhou F., Meng D., Wang Z. (2025). Segearth-ov: Towards training-free open-vocabulary segmentation for remote sensing images. Proceedings of the IEEE/CVF Conference on Computer Vision and Pattern Recognition.

[24] He K., Gkioxari G., Dollár P., Girshick R. (2017). Mask r-cnn. Proceedings of the IEEE International Conference on Computer Vision.

[25] Wang X., Kong T., Shen C., Jiang Y., Li L. (2020). Solo: Segmenting objects by locations. Proceedings of the European Conference on Computer Vision.

[26] Bolya D., Zhou C., Xiao F., Lee Y.J. (2019). Yolact: Real-time instance segmentation. Proceedings of the IEEE/CVF International Conference on Computer Vision.

[27] Liu S., Qi L., Qin H., Shi J., Jia J. (2018). Path aggregation network for instance segmentation. Proceedings of the IEEE Conference on Computer Vision and Pattern Recognition.

[28] Tian Z., Shen C., Chen H. (2020). Conditional convolutions for instance segmentation. Proceedings of the European Conference on Computer Vision.

[29] Peng S., Jiang W., Pi H., Li X., Bao H., Zhou X. (2020). Deep snake for real-time instance segmentation. Proceedings of the IEEE/CVF Conference on Computer Vision and Pattern Recognition.

[30] Liu Z., Liew J.H., Chen X., Feng J. (2021). DANCE: A Deep Attentive Contour Model for Efficient Instance Segmentation. Proceedings of the IEEE/CVF Winter Conference on Applications of Computer Vision.

[31] Wei S., Zhang T., Ji S. (2021). A Concentric Loop Convolutional Neural Network for Manual Delineation-Level Building Boundary Segmentation from Remote-Sensing Images. IEEE Trans. Geosci. Remote Sens..

[32] Wang L., Wang G., Luo X., Wang L., Yu W., Zhang Z., Gao H. (2025). Contour-based instance segmentation method of road scene. Sci. Rep..

[33] Xiao X., Wang K., Zhong Z., Qu W., Wu W., Cui Z., Su Y., Li A., Gong J., Li D. (2025). A novel data-driven based high-precision building roof contour full-automatic extraction and structured 3D reconstruction method combining stereo images and LiDAR points. Int. J. Digit. Earth.

[34] Yao W., Li C., Xiong M., Dong W., Chen H., Xiao X. (2025). ContourFormer: Real-Time Contour-Based End-to-End Instance Segmentation Transformer. arXiv.

[35] Zhang F., Nauata N., Furukawa Y. (2020). Conv-MPN: Convolutional Message Passing Neural Network for Structured Outdoor Architecture Reconstruction. Proceedings of the IEEE/CVF Conference on Computer Vision and Pattern Recognition.

[36] Newell A., Yang K., Deng J. Stacked Hourglass Networks for Human Pose Estimation. Proceedings of the European Conference on Computer Vision.

[37] Wu W., Qian C., Yang S., Wang Q., Cai Y., Zhou Q. (2018). Look at Boundary: A Boundary-Aware Face Alignment Algorithm. Proceedings of the IEEE Conference on Computer Vision and Pattern Recognition.

[38] Acuna D., Ling H., Kar A., Fidler S. (2018). Efficient Interactive Annotation of Segmentation Datasets with Polygon-RNN++. Proceedings of the IEEE Conference on Computer Vision and Pattern Recognition.

[39] Zhang Z., Li Z., Bi N., Wang J., Zhang S. (2019). PPGNet: Learning Point-Pair Graph for Line Segment Detection. Proceedings of the IEEE/CVF Conference on Computer Vision and Pattern Recognition.

[40] Hamaguchi R., Hikosaka S. (2018). Building Detection from Satellite Imagery Using Ensemble of Size-Specific Detectors. Proceedings of the IEEE Conference on Computer Vision and Pattern Recognition Workshops.

[41] Zhang R., Zhang Q., Zhang G. (2023). SDSC-UNet: Dual Skip Connection ViT-Based U-Shaped Model for Building Extraction. IEEE Geosci. Remote Sens. Lett..

[42] Zhou Y., Qi H., Ma Y. (2019). End-to-End Wireframe Parsing. Proceedings of the IEEE/CVF International Conference on Computer Vision.

